# Pre-pandemic cognitive function and COVID-19 mortality: prospective cohort study

**DOI:** 10.1007/s10654-021-00743-7

**Published:** 2021-04-24

**Authors:** George David Batty, Ian J. Deary, Catharine R. Gale

**Affiliations:** 1 Department of Epidemiology and Public Health, University College London, 1-19 Torrington Place, London, WC1E 6BT UK; 2 Lothian Birth Cohorts, Department of Psychology, University of Edinburgh, Edinburgh, UK; 3 Medical Research Council Lifecourse Epidemiology Unit, University of Southampton, Southampton, UK

**Keywords:** COVID-19, Cognitive function, Cohort

## Abstract

Poorer performance on standard tests of pre-morbid cognitive function is related to an elevated risk of death from lower respiratory tract infections but the link with coronavirus (COVID‑19) mortality is untested. Participants in UK Biobank, aged 40 to 69 years at study induction (2006–10), were administered a reaction time test, an indicator of information processing speed, and also had their verbal-numeric reasoning assessed. Between April 1st and September 23rd 2020 there were 388 registry-confirmed deaths (138 women) ascribed to COVID-19 in 494,932 individuals (269,602 women) with a reaction time test result, and 125 such deaths (38 women) in the subgroup of 180,198 people (97,794 women) with data on verbal-numeric reasoning. In analyses adjusted for age, sex, and ethnicity, a one standard deviation slower reaction time was related to a higher rate of death from COVID-19 (hazard ratio; 95% confidence interval: 1.18; 1.09, 1.28), as was a one standard deviation disadvantage on the verbal-numeric reasoning test (1.32; 1.09, 1.59). While there was some attenuation in these relationships after adjustment for additional covariates which included socio-economic status and lifestyle factors, the two pre-pandemic indicators of cognitive function continued to be related to COVID-19 mortality.

**Supplementary Information:**

The online version contains supplementary material available at 10.1007/s10654-021-00743-7.

## Introduction

Cognitive function—also referred to as mental ability or intelligence—refers to psychological functions that involve the storage, selection, manipulation, and organisation of information, and the planning of actions. Evidence from well-characterised cohort studies suggest that lower scores on standard tests of cognition are linked to elevated rates of premature mortality and cardiovascular disease [1]. Similar inverse relations have recently been reported for cognition and death from respiratory infection, comprising pneumonia and influenza [2]. Various explanations have been advanced, including the observation that people with higher cognitive ability are better equipped to obtain, process, and respond to disease prevention advice, as well as having healthier behaviours which include a lower prevalence of cigarette smoking [3], itself a risk factor for pneumonia [4].

These observations inevitably raise the possibility that individuals with lower mental ability may experience a higher burden of COVID-19. In a recent examination of primary care records, relative to an unaffected group, people with intellectual disability experienced around ten times the risk of death ascribed to COVID-19 and five times the risk of hospitalisation, even after adjustment for age and other co-morbidities [5]. In a field-based cohort study of a non-disabled population, of a range of baseline psychosocial factors, cognitive function was the most strongly predictive of hospitalisation for COVID-19, whereby a doubling of disease risk was apparent in the lowest scoring group [6]. With the pandemic evolving since we published those analyses using UK Biobank [6], there is now a sufficiently high number of deaths in this study to examine if similar results are apparent for pre-pandemic cognitive function and COVID-19 mortality—an association which, to the best of our knowledge, has yet to be tested.

## Methods

Between 2006 and 2010, baseline data were collected in the UK Biobank, a prospective cohort study. Conducted across 22 research assessment centres, a total of 502,655 individuals aged 40 to 69 years participated (response 5.5%) [7]. Ethical approvals were received from the North-West Multicentre Research Ethics Committee. Data are publicly available upon application (https://www.ukbiobank.ac.uk/), and the present analyses of anonymised data did not require further ethical permissions.

### Assessment of cognitive function

We use two tests of cognitive function as administered at baseline. Verbal and numeric (‘verbal-numeric’) reasoning was assessed using a computerized 13-item multiple-choice test with a two-minute time limit. The score derived was the number of correct answers. This test was introduced during the baseline assessment period; therefore, data are available for a subset of study members only (N = 180,914). Reaction time, which captures speed of information processing, is a knowledge-reduced indicator of cognition. Measured in the present study using a computerized Go/No-Go “Snap”-type game (N = 496,882), participants were presented with electronic images of two cards. If symbols on the cards were identical, participants were instructed to immediately push the button-box using their dominant hand. The first five pairs were used as a practice with the remaining seven pairs, containing four identical cards, forming the assessment. Reaction time score was the mean time (milliseconds) taken to depress the button after each of these four matching pairs was presented. Reaction time correlates with cognitive tests that involve complex reasoning and knowledge such that people with higher cognitive ability tend to have faster reaction times [8].

### Assessment of confounding factors

Covariates were also assessed at baseline. Socioeconomic status was quantified using self-reported educational qualifications (degree, other qualifications, no qualifications), occupational classification based on current job, and the Townsend index of neighbourhood deprivation (higher scores denote greater disadvantage). Ethnicity was categorised as White, Asian, Black, Chinese, Mixed, or ‘other’ ethnic group. Vascular or heart problems, diabetes, chronic bronchitis or emphysema, and asthma, were based on self-report of a physician diagnosis. Hypertension was defined as systolic/diastolic blood pressure ≥ 140/90 mmHg and/or self-reported use of antihypertensive medication. Study members were also asked whether they had ever been under the care of a psychiatrist for any mental health problem. C-reactive protein, glycated haemoglobin, and high-density lipoprotein cholesterol concentrations were based on assays of non-fasting venous blood. Height, weight–from which body mass index was computed–and forced expiratory volume in one second were measured using standard protocols. Cigarette smoking, physical activity, and alcohol consumption were assessed using standard enquiries. Study members were linked to national mortality records and death from COVID-19, our outcome of interest, was denoted by the emergency ICD-10 code U07.1 (COVID-19, virus identified).

### Statistical analyses

To summarise the relation between cognition and mortality we used Cox regression to compute hazard ratios with accompanying 95% confidence intervals. In these analyses we calculated effect estimates for tertiles of scores for both the test of verbal-numeric reasoning (< 4 [most disadvantaged], 5–6, ≥ 7) and reaction time (≤ 499 ms, 500–581, ≥ 582 [most disadvantaged]). We also computed hazard ratios for a unit (standard deviation) disadvantage in score for verbal numeric reasoning (per 2.16 point decrease) and reaction time (per 118.2 ms increase). The most basic analyses were adjusted for known COVID-19 risk factors (age, sex, and ethnicity). Retaining these covariates, we then explored the impact of separately controlling for socioeconomic circumstances, existing medical conditions, lifestyle factors, and biological indices.

## Results

In 502,655 study members (273,472 women), 404 deaths (145 in women) were ascribed to COVID-19 between April 1st and September 23rd 2020. In Table 1, in age-, sex- and ethnicity-adjusted analyses, we show, individually, the relation of the covariates and cognitive data with death from COVID-19. Twenty covariates were related to a higher risk of death from COVID-19 in these minimally-adjusted analyses; only the point estimate for regular intake of alcohol and asthma did not achieve statistical significance at conventional levels. Thus, there was a raised risk of COVID-19 death in people who were older, male, of ethnic minority ancestry, and socioeconomically disadvantaged. Those study members who smoked, reported less physical activity, and had higher body mass index, those with extant illness at baseline, and those with unfavourable levels of known cardiovascular disease biomarkers—lower lung function, higher systemic inflammation, lower high-density lipoprotein, and higher glycated haemoglobin—also experienced elevated rates of COVID-19 mortality.

**Table 1:** Cognitive function and covariates at baseline (2006–2010) according to death from COVID-19 (2020)

	COVID-19 mortality		*p* value	Hazard ratios (95% CI)
	Yes (n = 404)	No (n = 502,251)		
*Demographic factors*				
Age, year, mean (SD)	62.7 (6.03)	56.5 (8.09)	< 0.0001	3.07 (2.67, 3.53)
Female, N (%)	145 (35.9)	273,327 (54.4)	< 0.0001	0.48 (0.38, 0.59)
Non-white ethnicity	37 (9.27)	26,997 (5.41)	0.001	3.75 (2.67, 5.27)
*Comorbidities*				
Vascular or heart disease, N (%)	216 (54.3)	149,139 (29.8)	< 0.0001	1.79 (1.46, 2.19)
Hypertension, N (%)	313 (79.9)	282,324 (57.2)	< 0.0001	1.70 (1.32, 2.19)
Diabetes, N (%)	63 (15.8)	26,345 (5.27)	< 0.0001	2.31 (1.75, 3.06)
Chronic bronchitis or emphysema, N (%)	18 (4.46)	8,334 (1.66)	< 0.0001	2.37 (1.47, 3.80)
Asthma, N (%)	34 (8.42)	57,879 (11.5)	0.050	0.80 (0.56. 1.14)
Mental health—Psychiatric consultation, N (%)	56 (14.1)	57,625 (11.6)	0.117	1.42 (1.07, 1.88)
*Lifestyle factors*				
Current smoker, N (%)	50 (12.6)	52,580 (10.5)	< 0.0001	1.88 (1.37, 2.59)
No physical activity, N (%)	55 (14.0)	32,804 (6.63)	< 0.0001	2.54 (1.90, 3.40)
Drinks alcohol daily/almost daily, N (%)	85 (21.3)	101,707 (20.3)	0.641	0.88 (0.69, 1.12)
Body mass index, kg/m^2^, mean (SD)	29.7 (5.83)	27.4 (4.80)	< 0.0001	1.51 (1.39, 1.65)
*Biomarkers*				
Lung function, L, mean (SD)	2.53 (0.87)	2.81 (0.80)	< 0.0001	0.69 (0.60, 0.80)
C-reactive protein, mg/L, median (IQR)	1.72 (0.83, 3.51)	1.26 (0.63, 2.49)	0.0001	1.26 (1.12, 1.41)
High-density lipoprotein, mmol/L, median (IQR)	1.26 (1.09, 1.52)	1.40 (1.17, 1.67)	0.0001	0.79 (0.69, 0.90)
HbA1C, mmol/mol, median (IQR)	36.7 (33.8, 40.7)	35.2 (32.8, 37.9)	0.0001	1.25 (1.16, 1.35)
*Socioeconomic factors*				
No university education, N (%)	310 (80.7)	330,988 (67.3)	< 0.0001	1.75 (1.35, 2.26)
Neighbourhood deprivation score, median (IQR)	−0.887 (−3.04, −2.48)	−2.14 (−3.64, 0.55)	0.0001	1.45 (1.33, 1.59)
Personal service, sales occupations etc., N (%)	54 (27.8)	66,160 (19.0)	0.002	1.57 (1.15, 2.16)
*Cognitive factors*				
Verbal-numeric reasoning, mean (SD)	5.32 (2.20)	6.02 (2.16)	0.0002	1.32 (1.09, 1.59)
Reaction time, msec, mean (SD)	606.1 (136.4)	559.9 (118.2)	< 0.0001	1.18 (1.09, 1.28)

Hazard ratios are adjusted for age, sex, and ethnicity and expressed per category, or per SD increase for continuous variables (except for reasoning which is expressed per SD decrease [disadvantage]). The maximum analytical sample of 502,655 people was lower in selected analyses owing to missing data

In analyses adjusted for age, sex, and ethnicity, individuals with lower verbal-numeric reasoning scores had a higher risk of death ascribed to COVID-19 (hazard ratio per SD disadvantage; 95% confidence interval: 1.32; 1.09, 1.59) (Tables 1 and 2). Whereas adjusting for markers of socioeconomic position and biological factors had little impact on these effect estimates, adding co-morbidity to the multivariable model led to some attenuation (1.25; 1.04, 1.52). The greatest degree of attenuation, however, was evident after adjusting for lifestyle indices (1.16; 0.96, 1.41) which included smoking and body weight.

**Table 2:** Hazard ratios (95% confidence intervals) for the association of measures of baseline cognitive function (2006–2010) with death from COVID-19 (2020)

	Adjusted for age, sex, and ethnicity	Adjusted for age, sex, ethnicity, and comorbidity	Adjusted for age, sex, ethnicity, and markers of socioeconomic status	Adjusted for age, sex, ethnicity, and lifestyle factors	Adjusted for age, sex, ethnicity, and biological factors	Adjusted for all covariates
Verbal-numeric reasoning						
*Cases/Number at risk*	*125/180,198*	*123/177,361*	*90/145,975*	*119/177,545*	*77/126,190*	*58/101,731*
1 (most disadvantaged)	2.04 (1.30, 3.20)	1.86 (1.18, 2.93)	2.22 (1.26, 3.94)	1.52 (0.95, 2.43)	2.32 (1.30, 4.14)	2.33 (1.16, 4.69)
2	1.35 (0.86, 2.14)	1.29 (0.81, 2.03)	1.34 (0.78, 231)	1.27 (0.80, 2.01)	1.62 (0.90, 1.89)	1.39 (0.73, 2.67)
3	1.0 (reference)	1.0	1.0	1.0	1.0	1.0
P for trend	0.002	0.007	0.006	0.082	0.004	0.019
Per SD disadvantage	1.32 (1.09, 1.59)	1.25 (1.04, 1.52)	1.31 (1.02, 1.67)	1.16 (0.96, 1.41)	1.29 (1.01, 1.64)	1.27 (0.94, 1.71)
Reaction time						
*Cases/Number at risk*	*388/494,932*	*378/483,785*	*182/339,977*	*373/484,832*	*250/352,096*	*122/243,954*
1	1.0 (reference)	1.0	1.0	1.0	1.0	1.0
2	0.97 (0.73, 1.30)	0.92 (0.69, 1.24)	1.01 (0.67, 1.54)	0.94 (0.70, 1.27)	0.90 (0.62, 1.30)	0.84 (0.50, 1.43)
3 (most disadvantaged)	1.50 (1.15, 1.95)	1.40 (1.07, 1.83)	1.66 (1.13, 2.44)	1.35 (1.03, 1.77)	1.50 (1.08, 2.09)	1.75 (1.10, 2.77)
P for trend	0.001	0.003	0.004	0.009	0.003	0.006
Per SD disadvantage	1.18 (1.09, 1.28)	1.16 (1.07, 1.27)	1.21 (1.07, 1.37)	1.15 (1.06, 1.25)	1.19 (1.07, 1.31)	1.22 (1.04, 1.43)

Comorbidities: diagnoses of vascular or heart disease, diabetes, chronic bronchitis or emphysema, asthma, hypertension, and mental illness

Socioeconomic status (SES): educational attainment, occupational classification, and area deprivation. Lifestyle factors: body mass index, smoking status, alcohol intake, and physical activity. Biomarkers: Forced expiratory volume in the first second, and blood concentrations of C-reactive protein, glycated haemoglobin, and high-density lipoprotein

Associations with COVID-19 mortality are also shown by tertiles of the cognitive exposures in Table 2. In minimally-adjusted analyses (age, sex, ethnicity), relative to the highest performing tertile of verbal-numeric reasoning, those people in the lowest-scoring group experienced a doubling in the rate of death from COVID-19 (2.04; 1.30, 3.20). There was also evidence of a dose–response effect such that the intermediate cognition group (1.35; 0.86, 2.14) experienced an intermediate level of risk (P for trend: 0.002). Covariate control had little impact on these results, the exception being lifestyle factors. In those analyses, although rates of COVID-19 mortality were still associated with around a 50% increase risk in the lowest cognition-scoring group (1.52; 0.95, 2.43), statistical significance at conventional levels was lost.

Slower responders to the reaction time test had an elevated risk of death from the disease (hazard ratio per SD disadvantage: 1.18; 1.09, 1.28) (Tables 1 and 2). In contrast to the analyses for verbal-numeric reasoning, there was less attenuation of estimates after controlling for the same covariates (Table 2). Adjustment for lifestyle factors again resulted in the greatest attenuation of the cognition–mortality gradient but this was modest (1.15; 1.06, 1.25), and statistical significance at conventional levels was retained owing to the larger analytical sample in the reaction time analyses. Unlike the analyses of verbal-numeric reasoning, however, when the results were analysed by tertiles, there appeared to be a threshold effect in each model: only people in the slowest reaction time tertile had elevated risk; the hazard ratio in the intermediate group approximated 1. Collective control for all measured confounding factors did not impact further on the pattern of the relationship for death from COVID-19 and our two indicators of cognitive function.

Lastly, given the known correlation for education and verbal-numeric reasoning (r = 0.40, *p* < 0.0001, N = 178,908 in the present data), we conducted separate adjustment for education, one of three indicators of socioeconomic status used herein (Table 1a, appendix). Controlling for education had a marginal attenuating effect on the strength of the cognition–COVID-19 death association, whereas the addition of the other socioeconomic factors of area deprivation and occupational classification led to positive confounding such that the magnitude of categorical effects increased somewhat. It is also the case, however, that, owing to some missing data for occupation, the apparent difference in strength of the association may at least partly reflect differences in sample size. With reaction time being a knowledge-reduced measure of cognition, its relationship with education (r = 0.15, *p* < 0.0001, N = 487,993) was weaker than for verbal-numeric reasoning.

## Discussion

Our main finding was that, net of several covariates, poorer scores on two tests of cognition—verbal-numeric reasoning and reaction time—were associated with a higher risk of death from COVID-19. Patterns of attenuation were similar for both exposures such that the greater impact was apparent after control for lifestyle factors, whereas physiological indices had little if any attenuating effect. We also replicated known risk factors for death from COVID-19—being older, male, from an ethnic minority, socioeconomically disadvantaged, and having an extant somatic medical condition have been repeatedly linked with poor prognosis in COVID-19 patients in China [9] and elsewhere. Similarly, both verbal-numeric reasoning (hazard ratio per SD disadvantage; 95% confidence interval: 1.17; 1.14, 1.20) and reaction time (1.15; 1.14, 1.16) demonstrated the expected associations [1, 10] with all-cause mortality (31,187 deaths in the full cohort) (Table 1b, appendix). The raised risk of COVID-19 mortality seen in people with the most disadvantaged scores for reaction time—as described, a knowledge-reduced measure of cognition—corroborates the results for verbal-numeric reasoning which is more closely linked to education. It is also the case that the magnitude of association for scores on both tests were little attenuated after taking into account educational attainment.

### Plausible mechanisms

There has been a deluge of health advice in the current pandemic during an era when news outlets and social media platforms have never been more ubiquitous. Preventative information has ranged from the simple and practical to the complex, contradictory, false, and fraudulent. In order to diminish their risk of the infection, the population has to acquire, synthesise, and deploy this information but the ability to do so seems to vary by levels of health literacy [11] just as it may for its close correlate, cognitive function.

### Potential implications

People with low cognition scores may be conceptualised as being a vulnerable group. As well as seemingly having an elevated risk of death from COVID-19 in the present study, evidence suggests that people with lower ability report being less likely to take up the offer of a vaccine [12]. Lower cognitive function may therefore represent a dual burden, as is perhaps also the case for ethnic minority groups and socioeconomically disadvantaged communities [6]. Further, given the current overabundance of health opinion, governments and public health agencies have an increased responsibility to provide simple and practical advice. This has not always been so in the UK during the pandemic.

Lifestyle factors explained the greatest portion of the association between cognition and death due to COVID-19. As discussed, there is good evidence that people with lower ability have less favourable health behaviours—they are more likely to smoke, be physically inactive, have a less prudent diet, and drink heavily [3, 13–15]. The skills captured by cognition tests, such as comprehension and reasoning, may be important in the successful management of health behaviours. Behavioural modification advice could therefore be recalibrated to make it more suitable for people with lower ability.

### Study strengths and weaknesses

The strengths of our study include the well-characterised nature of the sample and the full coverage of the population for cause of death. Our study also has some weaknesses. With the present sample not being representative of the general UK population, death rates from leading causes and the prevalence of reported risk factors are known to be underestimates of those apparent in less select groups [7]; the same is likely to be the case for COVID-19 deaths. This notwithstanding, there is evidence that, for major causes of death, risk factor associations are externally valid [7]. Secondly, levels of our baseline data—exposures and covariates—will vary in the period between study induction in UK Biobank and the present pandemic. This is a perennial issue in cohort studies and one we were able to investigate using data from a resurveys that took place in a sub-sample 4 to 8 years after baseline examination. Analyses revealed moderate and similar levels of stability for verbal-numeric reasoning (r = 0.63, *p* < 0.001, N = 9689) and reaction time (r = 0.49, *p* < 0.001, N = 28,810) relative to key covariates such as such as cigarette smoking (0.60, *p* < 0.001, N = 31,037), blood pressure (0.65, *p* < 0.001, N = 19,772), and diabetes (r = 0.63, *p* < 0.001, N = 31,037). Most observational studies in the context of chronic disease epidemiology use a single baseline measurement of the exposure and relate it to disease onset. In the few analyses incorporating a repeat measurement, risk factor–disease associations appear to be strengthened [16] and we would anticipate the same for cognition. Lastly, as is inevitable, unmeasured confounding factors may offer additional explanatory power in this observational study.

In conclusion, in the present study, poorer performance on two pre-pandemic indicators of cognitive function, including reaction time, a knowledge-reduced measure, was related to death ascribed to COVID-19.

## Supplementary Information

Below is the link to the electronic supplementary material.

Supplementary file1 (DOCX 19 kb)
